# Demographic and clinical characteristics of participants in the Neuropsychiatric Genetics of African Populations-Psychosis (NeuroGAP-Psychosis) study: A descriptive analysis

**DOI:** 10.1371/journal.pone.0356360

**Published:** 2026-08-26

**Authors:** Rocky E. Stroud, Kimberly Hook, Melkam Alemayehu, Lukoye Atwoli, Mark Baker, Lori B. Chibnik, Bizu Gelaye, Stella Gichuru, Symon M. Kariuki, Karestan C. Koenen, Joseph Kyebuzibwa, Justin McMahon, Rehema M. Mwema, Carter P. Newman, Charles R. J. C. Newton, Linnet Ongeri, Dan J. Stein, Anne Stevenson, Solomon Teferra, Zukiswa Zingela, Dickens Akena

**Affiliations:** 1 Department of Epidemiology, Harvard T.H. Chan School of Public Health, Boston, Massachusetts, United States of America; 2 Stanley Center for Psychiatric Research, Broad Institute of MIT and Harvard, Cambridge, Massachusetts, United States of America; 3 Department of Psychiatry, School of Medicine, College of Health Sciences, Addis Ababa University, Addis Ababa, Ethiopia; 4 Department of Mental Health, School of Medicine, Moi University College of Health Sciences, Eldoret, Kenya; 5 Brain and Mind Institute, The Aga Khan University, Nairobi, Kenya; 6 Department of Medicine, Medical College East Africa, The Aga Khan University, Nairobi, Kenya; 7 Department of Neurology, Massachusetts General Hospital, Boston, Massachusetts, United States of America; 8 Chester M. Pierce, MD Division of Global Psychiatry, Massachusetts General Hospital and Department of Psychiatry, Harvard Medical School, Boston, Massachusetts, United States of America; 9 Epidemiology Branch, Division of Population Health Research, Division of Intramural Research, Eunice Kennedy Shriver National Institute of Child Health and Human Development, Bethesda, Maryland, United States of America; 10 Neurosciences Unit, Clinical Department, KEMRI-Wellcome Trust Research Programme-Coast, Kilifi, Kenya; 11 Department of Psychiatry, University of Oxford, Oxford, United Kingdom; 12 Department of Public Health, Pwani University, Kilifi, Kenya; 13 Department of Social and Behavioral Sciences, Harvard T.H. Chan School of Public Health, Boston, Massachusetts, United States of America; 14 Psychiatric and Neurodevelopmental Genetics Unit, Department of Psychiatry, Massachusetts General Hospital, Boston, Massachusetts, United States of America; 15 Department of Psychiatry, School of Medicine, College of Health Sciences, Makerere University, Kampala, Uganda; 16 Department of Psychiatry and Mental Health, University of Cape Town, Cape Town, South Africa; 17 Centre for Clinical Research, Kenya Medical Research Institute, Nairobi, Kenya; 18 SA MRC Unit on Risk and Resilience in Mental Disorders, University of Cape Town and Neuroscience Institute, Cape Town, South Africa; 19 Department of Psychiatry, Faculty of Medicine and Health Sciences, Stellenbosch University, Cape Town, South Africa; 20 Executive Dean’s Office, Faculty of Health Sciences, Nelson Mandela University, Gqebera, South Africa; University of Missouri School of Medicine, UNITED STATES OF AMERICA

## Abstract

**Background:**

Approximately 1.3 million individuals in sub-Saharan Africa, as well as millions of others worldwide, live with psychotic disorders. However, there is limited mental health research based on these populations and even less of a focus on neuropsychiatric genetics research within these settings.

**Methods:**

To address this gap, the Neuropsychiatric Genetics of African Populations-Psychosis (NeuroGAP-Psychosis), a case-control study, collected data identifying genetic and environmental risk factors associated with psychotic disorders in Ethiopia, Kenya, South Africa, and Uganda. This paper describes baseline demographic and clinical characteristics of the 42,953 participants recruited between 2018–2023 and compares these characteristics by country and case status.

**Results:**

Overall, 57.3% of participants were male (ranging from 65.8% in Ethiopia to 46.6% in Uganda). Among cases, the clinical diagnosis of schizophrenia or psychotic disorder not otherwise specified (NOS) ranged from 75.5% in Ethiopia to 44.4% in Uganda. Specific to bipolar disorder or mania NOS, the highest proportion was 53.9% in Uganda, and the lowest was 24.0% in Ethiopia. There were country-level and case-status differences across all demographic and clinical (e.g., physical comorbidities, substance use) variables.

**Conclusion:**

Many factors, including language of consent, recruitment catchment areas, diagnostic practices across countries, and translation and/or cultural interpretation of the measures used in this study may account for the differences observed across countries. These findings suggest the importance of ongoing evaluation of cross-national differences to explore clinical implications and patterns of symptoms amongst geographically and culturally diverse populations. Future analyses should evaluate within-country differences to better explore these variations.

## Introduction

Psychotic disorders are relatively uncommon but highly disabling conditions affecting more than 21 million people worldwide [1]. The burden of disease associated with these disorders is notable; for example, schizophrenia alone accounts for 13.4 million years of life lived with disability globally [1]. Individuals living with psychotic disorders are also at risk for premature mortality [2], as well as development of other comorbid physical health conditions (e.g., cardiovascular, metabolic, and respiratory diseases) [3–7]. Though limited, work to date across African countries has suggested lifetime prevalence of psychosis ranging from 1% to 4.4% [2]. Most persons living with psychotic disorders reside in low- and middle-income countries (LMICs), yet there is a substantial gap in mental health research from LMICs [8]. This is particularly true for genetic research on neuropsychiatric disorders, which has historically focused on individuals of European ancestry [9,10]. To address this divide, the Neuropsychiatric Genetics of African Populations-Psychosis (NeuroGAP-Psychosis) study sought to expand knowledge of the genetic and environmental risk factors for neuropsychiatric disorders in Africa through large-scale phenotyping, sample collection and analysis [9].

In the NeuroGAP-Psychosis study, cases were persons currently being treated for a psychotic disorder, including the diagnoses of schizophrenia, schizoaffective disorder, bipolar disorder, mania not otherwise specified (NOS), or psychosis not otherwise specified (NOS). These diagnoses were chosen for two reasons: (i) there is genetic correlation across these disorders (a correlation of 0.6 between schizophrenia and bipolar disorder, specifically [11]); and (ii) longitudinal studies show that specific psychotic disorder diagnoses are not stable and change over time in the life course [12]. NeuroGAP-Psychosis was one of the largest genetics studies of psychosis in African populations at its inception, and this broad definition allowed enrollment of participants with a wide range of psychotic symptoms and diagnoses.

The goals of this paper are to: (i) provide an updated overview of the NeuroGAP-Psychosis study design [9]; and (ii) summarize the baseline demographic and clinical characteristics across and between the participating countries. While NeuroGAP-Psychosis used a single methodological approach and common training techniques across all research staff, the results varied significantly between collection sites (in this paper, aggregated by country). Given the case-control study design, our results are not population-representative. However, we anticipate that this broad overview will provide insights into patterns observed in enrolled participants across all sites and will describe trends across sites comparing cases to controls.

## Methods

NeuroGAP-Psychosis is a case-control study (a full description of the study design can be found in the published protocol [9]) which collected genetic and phenotypic data across sites in Ethiopia, Kenya, South Africa, and Uganda. Cases included individuals currently being treated for a diagnosis of a psychotic disorder, while controls were participants present at medical facilities in the same geographic location with no history of a psychotic disorder. Cases and controls were balanced for age and sex.

Cases were eligible if they had chart review diagnosis of one of the following disorders: schizophrenia, schizoaffective disorder, bipolar disorder, mania NOS, or psychotic disorder NOS. Briefly, defining characteristics of schizophrenia include the presence of two or more of the following: delusions, hallucinations, disorganized speech, grossly disorganized or catatonic behavior and/or negative symptoms, resulting in impaired functioning in major life area (e.g., self-care). While schizoaffective disorder shares the characteristics described above with other psychotic disorders, a significant focus on the interplay between mood and psychosis differentiates schizophrenia and schizoaffective disorder, such that individuals with schizoaffective disorder must also experience a mood episode (i.e., major depressive or manic episode), delusions or hallucinations for two or more consecutive weeks without mood symptoms, and the presence of mood symptoms for the majority of the illness. Finally, diagnosis of bipolar disorder requires experience of a manic episode, which may present alongside major depressive episodes; psychotic features may or may not co-occur with bipolar disorder [13]. The “not otherwise specified” categories were used to capture subthreshold symptoms of psychotic disorders in the above categories.

Individuals included were those with a clinical diagnosis of psychosis (cases) as confirmed by local clinician referral and/or medical record review. As this study worked across four countries and dozens of different hospitals and clinics, diagnoses were based on the local expertise of the clinical teams in each setting and the diagnostic criteria used as standard in that community.

As detailed below, several updates were made to the original study design.

### Study sites

The sites listed in the original protocol included sites in Ethiopia (Amanuel Mental Specialized Hospital, Black Lion Hospital), Kenya (Moi Teaching and Referral Hospital and affiliated sites, Kilifi County Hospital, Malindi sub-County Hospital, Port Reitz sub-County Hospital, Coast General Teaching and Referral Hospital), South Africa (Valkenberg Hospital, Lentegeur Hospital, Western Cape community health clinics, Fort England Psychiatric Hospital, Elizabeth Donkin Hospital, Tower Psychiatric Hospital, Komani Hospital, Nelson Mandela Academic Hospital, Dora Nginza Hospital, Eastern Cape community health clinics), and Uganda (Butabika National Mental Health Referral Hospital, Naguru Regional Referral Hospital, Arua Regional Referral Hospital, Mbarara Regional Referral Hospital, Gulu Regional Referral Hospital). To expand recruitment and further diversify the sample set, ethical approvals were obtained to recruit in additional settings. In Ethiopia, we expanded recruitment to Jimma University Teaching Hospital and Zewditu Memorial Hospital, while in South Africa we expanded recruitment through the addition of Kimberly Mental Health Hospital and affiliated community health clinics in the Northern Cape province.

### Participant recruitment

Participants were recruited in one of 10 languages: Acholi-Luo, English, Luganda, Lugbara, or Runyankole (Uganda); Afrikaans, English, or isiXhosa (South Africa); Afaan Oromo or Amharic (Ethiopia); and English or Kiswahili (Kenya). All documents (consent and full phenotype battery) were translated into local languages and back translated into English, following standard best practices. Participants were recruited from the launch of the study in 01/02/2018 through the end of recruitment on 31/03/2023. More details about recruitment processes and procedures can be found in Stevenson et al [9].

### Inclusion and exclusion criteria

All participants were 18 years or older. Eligible cases were identified by study staff through medical record review. Detailed description of the inclusion and exclusion criteria can be found in the published protocol [9]. Following the launch of recruitment, study sites in South Africa and Ethiopia were granted ethical approval to recruit cases who were hospitalized (i.e., inpatient) at the time of enrollment due to a primary psychotic disorder, which previously had been an exclusion criterion [14]. Any participants who exhibited acute psychotic symptoms (i.e., were classified as inpatients in Kenya and Uganda and/or a statement from a physician saying that the individual was not stable enough to enroll in the study) were excluded. Current inpatient status for any reason remained an exclusion criterion at sites in Kenya and Uganda for the duration of the study. Participants who were hospitalized with acute symptoms due to alcohol or substance use continued to be excluded in all NeuroGAP-Psychosis collection sites.

### Training

For comprehensive details on the rigorous training approach established for this study, see Stevenson et al., 2019; briefly, this comprised standardized trainings across sites that include competencies such as recruitment and consenting; reliability collection of phenotype and genotype data; and weekly supervision among assessors, local teams, and lead site personnel. Significant attention was given to training to ensure the rigor of the data that was collected.

### Measures

Demographic information was collected from all participants including age, sex at birth, education, and marital status. Information on self-reported ethnicities and languages spoken was also collected. All participants completed the following measures: the University of California, San Diego Brief Assessment of Capacity to Consent (UBACC), a 10-item questionnaire that evaluated participant comprehension of study components [15]; the Alcohol, Smoking, and Substance Involvement Screening Test, version 3.0 (ASSIST), which screened for substance use over the past three months and across the lifespan (e.g., alcohol, tobacco, cannabis, khat) [16]; the Composite International Diagnostic Interview screener (CIDI), which captured chronic physical conditions (e.g., diabetes, HIV/AIDS) [17]; and the Life Events Checklist for Diagnostic and Statistical Manual of Mental Disorders-5 (LEC), a 17-item scale evaluating exposure to potentially traumatic events that can happen to you as a victim or as a witness, for a total of 32 response options [18]. Recent manuscripts from NeuroGAP-Psychosis study showed that the LEC has good construct validity within Ethiopia, Kenya, South Africa, and Uganda [19–22].

Prior to 2021, the Mini International Neuropsychiatric Interview, Standard 7.0.2 for Diagnostic and Statistical Manual of Mental Disorders-5 (MINI) [23] was administered to cases only; however, starting in 2021, both cases and controls received the MINI. Participants completed modules A, C, K, and O, which evaluated major depressive episodes, manic and hypomanic episodes, psychotic disorders, and mood disorders with psychotic features, respectively. Based on the modules administered, there were a total of 19 possible diagnoses. Of these, based on the NeuroGAP-Psychosis definition of psychosis (i.e., using an umbrella term to combine bipolar disorders and psychotic disorders), there were 16 possible MINI diagnoses (e.g., “any psychotic disorder, lifetime;” “bipolar disorder, past”) considered as a psychosis diagnosis. As described in Stevenson et al [9], there is evidence showing a high level of genetic correlation between these disorders [11], and we have applied that methodology to our phenotype definition. Evaluation of the diagnostic validity of the MINI across the different study sites is ongoing [24,25].

In addition to the above, controls completed the Psychosis Screening Questionnaire (PSQ) [26], a brief tool to evaluate psychotic symptoms; the PSQ reliably assessed for these symptoms across the sites [26–28]. Controls also completed the Kessler Psychological Distress Scale (K10) [29], a 10-item measure to screen for symptoms of anxiety and depression, which demonstrated good construct validity and internal consistency across Ethiopia, Kenya, South Africa, and Uganda (Cronbach’s alpha = 0.83, 0.85, 0.84, and 0.86, respectively) [10,30–32].

### Sample size

The original target sample size for NeuroGAP-Psychosis was 34,000 participants, with equal numbers of cases and controls. The recruitment goal was expanded to 43,000 participants.

### Genetic analysis

In response to developments by colleagues [33], the genetic analysis plan changed from genotyping using the Global Screening Array to a new technology developed at the Broad Institute for low-pass whole genome sequencing and blended genome exome sequencing [34]. Genomic data generation is ongoing.

### Statistical methods

The distribution of the baseline characteristics of the sample, including demographic and clinical measures, were analyzed overall and stratified by country. Means and standard deviations were presented for continuous data. Frequency distributions and percentages were calculated for categorical data.

Due to our large sample size statistical tests comparing across countries will produce p-values less than 0.001 for even small differences therefore, we present these results as descriptive and make no conclusions about significance. Analysis was done using SPSS version 28.

### Patient and public involvement

Patients and the public were not involved in the development of this study.

### Ethics

Ethical clearances for this study were obtained from all participating sites. Ethiopia: Addis Ababa University College of Health Sciences (#014/17/Psy) and the Ministry of Science and Technology National Research Ethics Review Committee (#3.10/14/2018); Kenya: Moi University School of Medicine Institutional Research and Ethics Committee (#IREC/2016/145, approval number: IREC 1727), Kenya National Council of Science and Technology (#NACOSTI/P/17/56302/19576), KEMRI Centre Scientific Committee (CSC# KEMRI/CGMRC/CSC/070/2016), KEMRI Scientific and Ethics Review Unit (SERU#KEMRI/SERU/CGMR-C/070/3575); South Africa: The University of Cape Town Human Research Ethics Committee (#466/2016) and Walter Sisulu University Research and Ethics Committee (# 051/2016); Uganda: The Makerere University School of Medicine Research and Ethics Committee (SOMREC #REC REF 2016−057) and the Uganda National Council for Science and Technology (UNCST #HS14ES); and USA: the Harvard T.H. Chan School of Public Health (#IRB17–0822).

Data were collected between February 2018 and March 2023. All participants provide written informed consent. Additional information regarding the ethical, cultural, and scientific considerations specific to inclusivity in global research is included in the Supporting Information documents.

## Results

Across all sites, 42,953 participants were recruited into the study between 2018 and 2023; of these, 21,618 were cases (50.3%), and 21,335 were controls (49.7%) (Table 1). Of note, our sample consists of individuals representing over 100 self-reported ethnicities across four African countries, showcasing the program’s success in enrolling a diverse sample. Results demonstrated notable differences between countries for all clinical characteristics reported. Below are results for the entire study by case status, as well as notable country-specific differences. Full results are reported in Tables 2 and 3.

**Table 1:** Demographic characteristics among cases and controls, aggregated and by country (total N = 42,953).

	All Sites			Ethiopia			Kenya			S. Africa			Uganda		
	All	Case	Control	All	Case	Control	All	Case	Control	All	Case	Control	All	Case	Control
N, %	*N* = 42,953	*21,618 (50.3)*	*21,335 (49.7)*	*N* = 13,000	*6,500 (50)*	*6,500 (50)*	*N* = 8,355	*4,415 (52.8)*	*3,940 (47.2)*	*N* = 9,598	*4,703 (49.0)*	*4,895 (51.0)*	*N* = 12,000	*6,000 (50.0)*	*6,000 (50.0)*
**Age (Years)**, mean (SD)	**36.7 (11.7)**	36.9 (11.6)	36.5 (11.8)	**36.6 (10.6)**	37.0 (10.3)	36.2 (10.9)	**35.9 (12.0)**	35.8 (12.0)	36.0 (12.0)	**38.7 (12.1)**	39.1 (11.8)	38.4 (12.3)	**35.7 (12.2)**	36.0 (12.2)	35.5 (12.2)
**Sex** (%)															
*Male*	**57.3**	57.9	56.8	**65.2**	65.8	64.7	**53.1**	54.1	52.0	**63.8**	65.1	62.5	**46.6**	46.6	46.6
*Female*	**42.7**	42.1	43.2	**34.8**	34.2	35.3	**46.9**	45.9	48.0	**36.2**	34.9	37.5	**53.4**	53.4	53.4
**Educational Status** (%)															
*No education or some primary school*	**19.6**	22.8	16.4	**25.7**	33.0	18.5	**12.6**	16.3	8.4	**8.6**	10.9	6.5	**26.8**	26.0	27.6
*Finished primary or some secondary school*	**37.5**	40.6	34.3	**24.2**	27.4	20.9	**30.8**	38.6	22.1	**55.1**	56.4	53.8	**42.5**	44.1	40.8
*Finished secondary or some college*	**26.6**	24.1	29.1	**26.0**	23.4	28.7	**33.2**	30.2	36.6	**27.8**	24.1	31.3	**21.7**	20.4	23.0
*Finished college or beyond*	**16.3**	12.4	20.2	**24.1**	16.2	32.0	**23.4**	14.9	33.0	**8.5**	8.6	8.5	**9.0**	9.5	8.5
**Living Arrangement (%)**															
*Lives alone*	**13.4**	10.5	16.3	**12.6**	11.6	13.7	**13.9**	9.6	18.8	**15.6**	10.5	20.6	**12.1**	10.0	14.1
*Lives with parental family*	**32.4**	41.3	23.4	**38.6**	45.8	31.3	**31.4**	43.0	18.5	**35.2**	46.9	23.9	**24.2**	30.8	17.6
*Lives with spouse or partner*	**35.0**	24.5	45.6	**38.4**	26.8	49.9	**39.4**	31.6	48.2	**24.0**	9.9	37.5	**37.1**	28.3	45.9
*Lives with other relatives and/or friends*	**18.7**	23.1	14.4	**10.1**	15.5	4.8	**15.1**	15.7	14.5	**23.8**	30.6	17.4	**26.5**	30.8	22.2
**Marital Status (%)**															
*Married or cohabitating*	**38.2**	28.3	48.2	**39.6**	28.2	50.9	**44.1**	36.1	53.0	**25.1**	12.6	37.1	**43.1**	35.0	51.3
*Widowed, divorced, separated, or annulled*	**15.8**	18.6	12.9	**13.3**	15.4	11.2	**16.6**	20.5	12.1	**11.7**	12.1	11.4	**21.2**	25.7	16.6
*Never married*	**46.0**	53.0	38.8	**47.1**	56.3	37.9	**39.4**	43.4	34.8	**63.1**	75.1	51.5	**35.7**	39.2	32.1
**Language of Consent (%)**															
*Acholi-Luo*	**5.0**	5.1	4.9	**–**	–	–	**–**	–	–	**–**	–	–	**17.9**	18.5	17.4
*Afrikaans*	**2.0**	2.0	2.1	**–**	–	–	**–**	–	–	**9.1**	9.3	9	**–**	–	–
*Amharic*	**29.1**	28.6	29.6	**96.1**	95.0	97.3	**–**	–	–	**–**	–	–	**–**	–	–
*English*	**28.6**	26.6	30.7	**–**	–	–	**38.1**	28.7	48.6	**42.8**	41.8	43.8	**41.7**	42.0	41.5
*isiXhosa*	**10.7**	10.7	10.8	**–**	–	–	**–**	–	–	**48.1**	49.0	47.2	**–**	–	–
*Kiswahili*	**12.0**	14.6	9.5	**–**	–	–	**61.9**	71.3	51.4	**–**	–	–	**–**	–	–
*Luganda*	**5.9**	5.4	6.4	**–**	–	–	**–**	–	–	**–**	–	–	**21.0**	19.4	22.6
*Lugbara*	**2.7**	2.8	2.6	**–**	–	–	**–**	–	–	**–**	–	–	**9.6**	10.1	9.2
*Oromo*	**1.2**	1.5	0.8	**3.9**	5	2.7	**–**	–	–	**–**	–	–	**–**	–	–
*Runyankole*	**2.7**	2.8	2.6	–	–	–	**–**	–	–	**–**	–	–	**9.7**	10.0	9.4

**Table 2:** Clinical characteristics among cases and controls, aggregated and by country (total N = 42,953).

		All Sites		Ethiopia		Kenya		S. Africa		Uganda	
		Case	Control	Case	Control	Case	Control	Case	Control	Case	Control
N		N = 21,618	N = 21,335	N = 6,500	N = 6,500	N = 4,415	N = 3,940	N = 4,703	N = 4,895	N = 6,000	N = 6,000
**Age (Years)**, mean (SD)		36.9 (11.6)	36.5 (11.8)	37.0 (10.3)	36.2 (10.9)	35.8 (12.0)	36.0 (12.0)	39.1 (11.8)	38.4 (12.3)	36.0 (12.2)	35.5 (12.2)
**UBACC**	Trial 1^a^, mean (SD)	12.5 (3.5)	14.0 (3.4)	14.6 (3.1)	16.4 (2.2)	10.8 (3.1)	12.6 (3.0)	12.3 (3.4)	13.9 (3.1)	11.8 (3.3)	12.5 (3.6)
	Trial 2, mean (SD)	16.3 (3.2)	17.4 (2.8)	17.9 (2.6)	19.0 (1.4)	14.5 (2.9)	16.1 (2.8)	16.7 (3.0)	18.0 (2.4)	15.4 (3.0)	16.0 (3.1)
**LEC**	Any trauma exposure, %	77.3	76.7	66.7	71.4	84.3	84.8	98.2	98.9	68.7	59.0
**ASSIST**	Any regular^b^ substance use, %	27.3	27.3	24.3	23.6	14.9	11.0	64.7	59.2	10.3	15.9
	Tobacco regular use, %	21.3	14.4	13.2	4.8	10.0	4.6	62.2	45.9	6.3	5.4
	Alcohol regular use, %	7.3	17.8	7.6	17.5	5.8	5.8	9.6	33.6	6.1	13.3
	Khat regular use, %	6.0	3.5	16.2	9.0	3.6	2.5	0.1	0.1	1.2	1.1
	Cannabis regular use, %	3.2	3.3	0.5	0.4	2.9	1.8	10.8	11.7	0.4	0.6
**CIDI**	Any physical comorbidity, %	50.7	64.2	36.7	56.5	47.7	57.2	59.1	74.2	60.5	68.9
	HIV, %	5.1	11.9	2.3	8.6	5.6	10.8	8.0	14.9	5.5	13.9
**PSQ**	Lifetime symptoms, %	–	8.8	–	2.4	–	7.7	–	13.6	–	12.3
	Current symptoms, %	–	4.0	–	1.6	–	2.5	–	6.5	–	5.5
**K10**	Low distress, (scores: 10–15), %	–	85.3	–	93.4	–	86.4	–	68.7	–	89.4
	Moderate distress, (scores: 16–21), %	–	10.4	–	4.8	–	10.5	–	21.1	–	7.8
	High distress, (scores: 22–29), %	–	3.4	–	1.5	–	2.4	–	8.3	–	2.3
	Very high distress, (scores: 30+), %	–	0.8	–	0.3	–	0.7	–	1.8	–	0.6
**Chart Review Diagnosis**	Schizophrenia or Psychotic Disorder Not Otherwise Specified, %	60.9	–	75.5	–	58.3	–	64.0	–	44.4	–
	Bipolar Disorder or Mania Not Otherwise Specified, %	35.5	–	24.0	–	36.9	–	26.6	–	53.9	–
	Schizoaffective Disorder, %	3.7	–	0.5	–	4.8	–	9.4	–	1.7	–
	Inpatient, %	15.5	–	12.2	–	–	–	47.7	–	–	–

Percentages may not add up to 100% due to missing information for some participants.

a Score of more than 14.5 needed by fourth trial to pass screening for decisional capacity making.

b Regular use is defined as reporting use of substance daily or weekly.

**Table 3:** MINI symptom endorsement and diagnoses among cases and controls, aggregate by country (total N = 28,518).

		All Sites		Ethiopia		Kenya		S. Africa		Uganda	
		Case	Control	Case	Control	Case	Control	Case	Control	Case	Control
		*N* = 21,436	*N = 7,082*	*N* = 6,416	*N = 2,303*	*N* = 4,394	*N = 682*	*N* = 4,682	*N = 1,806*	*N* = 5,944	*N = 2,291*
**Symptoms**	Endorsement of screening questions for depressive episodes, %	67.4	11.3	52.2	4.9	90.3	2.2	53.2	17.8	78.2	15.4
	Past suicidal ideation, intent, plan, or attempts, %	53.3	36.3	52.1	17.9	50.1	20.0	60.9	37.0	52.9	42.2
	Sample Size for suicidality question	(N = 14,454)	(N = 802)	(N = 3,351)	(N = 112)	(N = 3,967)	(N = 15)	(N = 2,490)	(N = 322)	(N = 4,646)	(N = 353)
	Endorsement of screening questions for manic episodes, %	68.8	5.3	44.5	1.1	84.3	0.6	69.4	15.7	83.0	2.8
	Endorsement of screening questions for psychotic episodes, %	95.0	11.6	96.2	1.9	98.4	1	91.5	28.7	93.9	11.1
**Psychotic disorder by type** ^**a**^	Any psychotic disorder, lifetime, %	36.7	1.9	44.4	0	9.8	0	44.9	4.5	42.0	2.2
	Mood disorder with psychotic features, lifetime, %	23.9	0.8	19.6	0	39.9	0.1	17.4	1.8	21.8	1.0
	Bipolar disorder with psychotic features, past, %	59.4	1.8	31.8	0.1	77.7	0.3	62.6	5.2	73.0	1.3
	Bipolar disorder, past, %	61.4	2.1	34	0.1	78.3	0.3	65.6	6.1	75.2	1.4
**Major depressive disorder, past,** %		13.1	4.9	19.7	1.0	13.7	0.3	6.2	7.8	10.9	7.8

The differences in total N for controls between Tables 2 and 3 occurred as the MINI was only administered to controls starting in 2021.

^a^Psychotic disorder types are not mutually exclusive.

The mean age for cases was 36.9 years (SD ± 11.6) and 36.5 years (SD ± 11.8) for controls. Males comprised the majority of cases (57.9%) and controls (56.8%). Controls were more highly educated with 20.2% completing college or beyond compared to 12.4% of cases. Most cases were never married (53.0%), while controls were more likely to be married (48.2%). Most cases lived with their parental family (41.3%), while most controls lived with a spouse/partner (45.6%). Among cases, Amharic (28.6%) and English (26.6%) were the most common languages of consent, followed by Kiswahili (14.6%) and isiXhosa (10.7%). Amharic, English, isiXhosa, and Kiswahili were also the most common consent languages for controls (29.6%, 30.7%, 10.8%, and 9.5% respectively) (Table 1).

Country-level differences were observed across demographic variables. Sites in Ethiopia and South Africa recruited male cases at higher proportion (65.8% and 65.1%, respectively), as compared to sites in Kenya and Uganda (54.1% and 46.6%, respectively). Sites enrolled male controls at similar proportions to create a balanced dataset within each country. In Ethiopia, South Africa, and Uganda, the distribution of language consent was similar between cases and controls; however, in Kenya, more cases elected to be recruited in Kiswahili (71%) as compared to controls (51%) (Table 1).

Across all countries, cases averaged a score of 12.5 (SD ± 3.5) and 16.3 (SD ± 3.2) on Trials 1 and 2 of the UBACC, respectively. Comparatively, controls averaged a score of 14.0 (SD ± 3.4) for Trial 1 and 17.4 (SD ± 2.8) for Trials 2 (Table 2).

### Determination of psychotic disorder

Among 21,618 cases, 60.9% were diagnosed with schizophrenia or psychotic disorder NOS; 35.5% were diagnosed with bipolar disorder or mania NOS; and 3.7% were diagnosed with schizoaffective disorder (Table 2). When collection sites were compared grouped by country, differences emerged regarding patterns in diagnoses. For example, among cases in Ethiopia, 75.5% were diagnosed with schizophrenia or psychotic disorder NOS versus only 44.4% of such cases from Uganda. In contrast, 53.9% of cases from Uganda were diagnosed with bipolar disorder or mania NOS versus 24.0% of cases from Ethiopia (Table 1).

Among cases with confirmed psychotic disorder on medical record review, 80.5% met criteria for any psychotic disorder on the MINI. In contrast, among controls, only 3.8% met criteria for a psychotic disorder on the MINI (Table 3). Using the PSQ, which was only administered in controls, 4.0% and 8.8% of controls reported current and lifetime psychotic symptoms, respectively (Table 2).

### Clinical characteristics

More than one quarter of both cases and controls (27.3%) reported any regular substance use, defined as use of a substance daily or weekly, with the most common frequently used substance differing between cases and controls (tobacco for cases (21.3%) and alcohol for controls (17.8%)). We observed variations in type of substance use across countries, such that khat was the most commonly used substance for cases in Ethiopia, as compared to tobacco in all other sites. Similarly, 64.7% of cases and 59.2% of controls from South Africa reported regular substance use on the ASSIST versus 10.3% of cases and 15.9% of controls in Uganda (Table 2).

Over half of cases (50.7%) reported at least one physical comorbidity on the CIDI, compared to nearly two-thirds of controls (64.2%) (Table 2).

Cases and controls were similar in reporting exposure to at least one potentially traumatic event on the LEC (77.3% and 76.7%, respectively). From our data, the highest endorsement occurred in South Africa, in which 98.2% of cases and 98.9% of controls reported one or more exposure to a potentially traumatic event on the LEC versus 66.7% of cases (Ethiopia) and 59.0% of controls (Uganda), which had the lowest endorsement levels by case status. Table 1 compares endorsements by country and case status across several tools.

While most controls reported low psychological distress on the K10 (85.3%), a number of controls disclosed moderate (10.4%) to high (3.4%) to very high (0.8%) distress (Table 2).

Using the MINI, the vast majority of cases (80.5%) reported a history of psychotic symptoms and received a psychosis diagnosis. In contrast, nearly all controls were individuals without a psychosis diagnosis (96.2%) on the MINI. The MINI captured similar endorsements of psychosis in Kenya, South Africa and Uganda (85.7%, 82.6% and 86.6%, respectively), identifying approximately 85% of individuals with a diagnosis of a psychotic disorder from chart review; however, in Ethiopia, only 69.8% of cases received a confirmatory diagnosis on the MINI.

Of the cases who received the MINI (N = 21,436, 99.2% of cases), 67.4% reported past depressive symptoms, and 13.1% of the sample met the criteria for past major depressive disorder. In the MINI Module A (Major Depressive Episode), if participants did not endorse at least one of the two screening questions, the participants were skipped out of the rest of the module. Thus, only 67.4% of cases (N = 14,454) were administered a question regarding suicidality. Over half (53.3%) of this subset endorsed past suicidal ideation, intent, plan, or attempt. Of the controls who received the MINI (N = 7,082, 33.2%), 11.3% reported past depressive symptoms, and 4.9% met the criteria for past major depressive disorder. Again, due to the MINI’s branching logic, only 11.3% of controls (N = 802) were administered a question on suicidality. Approximately one third (36.3%) of controls from this subset endorsed past suicidal ideation, intent, plan, or suicidal attempt.

## Discussion

The NeuroGAP-Psychosis study is the largest neuropsychiatric genetics research program in Africa to date, as well as the largest study of psychotic disorders ever conducted on the continent. The broad goal of the study was to address the lack of representation for African populations in neuropsychiatric genetics research with a particular focus on psychosis.

This descriptive analysis of data from NeuroGAP-Psychosis displayed three key findings. First, control participants were recruited from the same underlying population as case participants, that is from healthcare facilities in the same geographic areas and balanced with cases based on age and sex. Controls were at the medical facility for a variety of different reasons, including individuals seeking care, persons acting as caregivers for someone else, or those who were staff members or students. The decision to recruit from healthcare facilities enabled this study to enroll large numbers of controls with low frequency of psychopathology; however, as controls were recruited from healthcare facilities (and most controls were receiving medical care at the time of study enrollment), a large proportion endorsed physical health problems. Second, despite using the same study design and rigorous implementation and quality control standards, we found notable differences in case characteristics across countries. Third, the heterogeneity across countries and settings means that study-wide, aggregate data allows for larger sample size comparisons, though stratification by country is needed to understand the factors specific to each site.

### Study design and recruitment contributed to heterogeneity across countries and limited case-control comparisons

Tables 1–3 display data in two ways: cases and controls (i) in aggregate for the entire study sample and (ii) stratified by country. There are wide-ranging differences among demographic and clinical characteristics by country, which are key factors for exploration. For this reason, we highlight the importance of looking at aggregate results while evaluating how country-specific differences may drive these numbers. The primary aim of the NeuroGAP-Psychosis study was to investigate the genetic underpinnings of psychosis in African populations, while also enrolling participants in a large-scale effort. Efforts to achieve this aim resulted in heterogeneity by country in diagnostic groups, risk factors, and underlying sample populations.

Cases and controls were both recruited from health facilities in similar geographic regions. Cases were predominantly recruited from mental health hospitals or during mental health clinic days, and controls were recruited from outpatient general health facilities (whether they were themselves patients, caregivers or staff). This approach had practical implications for control recruitment: i) teams were able to approach and enroll participants in vast numbers in a relatively short amount of time; ii) teams had access to patient medical charts to gather additional information on medications and family medical histories, ruling out frank psychopathology for controls; and iii) study staff were able to make referrals directly to on-call medical staff when risk of harm protocols were triggered from incidental findings learned about during study procedures.

It should be noted that as recruitment was undertaken at healthcare facilities, a significant proportion of controls reported physical health problems, and these rates cannot be assumed to be representative of the broader population. Similarly, due to selection bias from enrolling participants from healthcare settings, comparisons between cases and controls cannot be directly made or assumed to be representative of the general population. For example, in the entire sample and when stratified by country, controls had a higher prevalence (64.2%) of physical health problems than cases (50.7%). This is a deviation from the well-established literature, which generally notes a trend of higher physical health comorbidities among individuals with psychosis [5]. Finally, there were some people recruited into the study as controls who may have had undiagnosed mental health disorders and/or individuals who endorsed possible mental health symptoms on the self-reported phenotype tools that flagged possible diagnoses (of note, the percentages of controls who potentially endorsed symptoms of psychotic disorders were very low). We did not consider this to be a significant source of error as there is a very low number of such individuals in the controls category, and we also know that 1) some people may endorse certain symptoms of disorders and yet not meet diagnostic threshold, and 2) these individuals who did endorse symptoms were not formally assessed by a physician as in our population of cases.

### Study-wide comparisons versus country-specific comparisons

In Kenya, South Africa and Uganda, there was a high rate of confirmatory diagnosis of a psychosis diagnosis for cases (85.7%, 82.6% and 86.6%, respectively); however, in Ethiopia, the rate was lower at 69.8%. Some potential explanations for these rates across all sites include i) patients at the time of enrollment were either in full or partial remission which resulted in sub-threshold symptom endorsement; and ii) patient recall of their worst psychotic episode may be limited. While all research staff across all four countries were trained on the administration of the MINI in a standardized way, and participants were recruited using the same methods, there are several possibilities that may account for the difference in the confirmatory diagnosis from the MINI. One example is that no participants completed the study procedures in English in Ethiopia, and translation and/or cultural interpretation may have affected items endorsed on the MINI, as discussed above. This is in contrast to the over 40% of case participants who completed study procedures in English in South Africa and Uganda, where English is one of the national languages.

As expected, after the MINI was implemented with control participants, very few (roughly 4%) of controls received a psychosis diagnosis on the MINI or endorsed any manic or psychotic symptoms (~15%).

NeuroGAP-Psychosis planned to recruit an equal number of male and female participants in both cases and controls. However, in Ethiopia and South Africa, the ratio of male-female recruitment of cases neared 2:1, while in Kenya and Uganda, the male-female case ratio was closer to 1:1. Controls were enrolled in the same male to female ratio as the cases to ensure balance. Previous studies have found sex differences in the prevalence of psychotic disorders [35–37]. However, due to the study design, inferences cannot be made about prevalence patterns by sex. Rather, given that all participants were recruited in healthcare settings, it is possible that selection factors explain the differences observed. For example, participants were recruited from large referral psychiatric hospitals or clinics, which tend to be heavily populated with male patients [35,37,38]. Specifically, there are eight male wards at Amanuel Hospital in Addis Ababa, Ethiopia compared to its four female wards, while the ratio of male-female wards at Butabika Referral Hospital in Kampala, Uganda is 4:3. As these large referral hospitals typically receive patients exhibiting the most severe psychiatric symptoms, and men often demonstrate more visible symptoms, females with psychotic disorders are often undetected [39] and are thus less likely to be hospitalized.

Across countries, we observed different frequencies of diagnoses obtained in participants’ medical charts; for example, bipolar disorder diagnoses from participants’ charts varied from about a quarter in Ethiopia and South Africa to about one third in Kenya and roughly half in Uganda. While it is possible that there are different prevalences of psychotic disorders among our clinical recruitment sites, these same patterns were not observed on other measures used in the study (i.e., MINI). Other factors that may account for these patterns across chart review diagnoses might be attributed to differences in diagnostic practices stemming from cross-cultural variations about the perceptions of symptoms and disorder classification [40] or impact of sex differences on diagnostic patterns such as described above [39].

Language, and the use of ten different languages to recruit, enroll, and administer questionnaires to participants in this study, could also partially account for differences within and between countries. English, the original language of the measures used in this study, was a language option in all four study countries; however, no participants in Ethiopia chose to complete study procedures in English, while nearly half of participants completed the study in English in Kenya, South Africa and Uganda. It is noteworthy that English is not an official language in Ethiopia. Even after following best practice procedures for cultural adaptation and contextual translation in this study, there is a possibility that contextual translation alone did not fully capture cross-cultural variations in mental health constructs and description of psychiatric symptoms [41,42]. This potentially impacted item endorsement on measures and patterns seen in our results.

### Study-wide comparisons versus country-specific comparisons: Clinical factors

The MINI Module A for Major Depressive Disorder has two initial screening questions for depressive symptoms. The endorsement of these symptoms by cases across all four countries was high, particularly in Kenya (over 90%). Despite this, most participants did not endorse the full range of symptoms needed to meet criteria for a full diagnosis of major depressive disorder on the MINI. While some depressive symptoms may have been captured under MINI diagnoses of bipolar disorder or other mood disorders, the observed low frequency of major depressive disorder were nevertheless unexpected, as depression and psychotic disorders are often comorbid [43,44]. We also observed very low proportion of depressive symptoms among control participants, particularly in Ethiopia and Kenya (4.9% and 2.2%, respectively). Similarly, we observed very low proportion of controls meeting criteria for major depressive disorder on the MINI, particularly in Ethiopia and Kenya (1.0% and 0.3%, respectively). This is in contrast to estimates from other studies, including past work from Ethiopia, that suggest the prevalence of depression is 9.1% [45], or typical estimates in general populations [46–48] and among populations with higher proportion of chronic conditions [49].

Across countries, endorsement of exposure to potentially traumatic events was consistently high; for example, in South Africa, nearly the entire sample reported exposure to at least one potentially traumatic event that directly happened to them or that they witnessed. The prevalence of trauma exposure was roughly identical across participants by case and control status. The prevalence of trauma exposure captured in this study is consistent with those from prior studies [50–53] and underscores the need for ongoing investigation into the burden of these traumatic events and their implications for physical and mental health.

As expected, patterns of substance use varied within and between countries, though regular substance use did not seem to be associated with case status. However, there were differences among the most frequently used substances: cases were more likely to use tobacco, whereas controls were more likely to use alcohol. High tobacco use among individuals with psychotic disorders has been identified in previous literature [54,55], including in South Africa [56]. Different patterns also existed on a country level, which may reflect regional preferences or cultural norms. Specifically, among study sites, cannabis is legalized for personal use only in South Africa. Additionally, khat is used primarily in East Africa, and our sample accordingly found the highest prevalence of khat use in Ethiopia, followed by Kenya. In these countries, khat use was higher among cases, and khat has previously been associated with psychosis symptoms [57].

### Limitations

It is important to note the differences represented in this study among our collection sites across Ethiopia, Kenya, South Africa and Uganda and that any findings at the country-level in this paper are not generalizable or representative of that particular country. Variations in reported prevalence rates could also be impacted by factors such as differing approaches to measuring psychiatric symptoms and varying cultural presentation of mental health symptoms. Delving into these potential variations is beyond the scope of the current manuscript, as it was not possible to document all of these factors across four countries and dozens of clinics.

Additionally, in order to ensure the ability of case and control participants to adequately consent to enrollment in the study, participants were required to score above 14.5 on the UBACC. This may have excluded participants with low education, cognitive impairment, or other factors that may affect UBACC performance.

## Conclusion

Given that these results showcased such differences within and among these four African countries, it is vital that future research delves deeper into evaluating cross-national differences to explore clinical implications and patterns of symptoms amongst geographically and culturally diverse populations, as these differences may have profound impacts on the diagnosis and treatment of individuals with psychotic disorders. Continuing to invest in work that adapts and validates instruments to ensure they are maximally context specific, sensitive, and relevant is one important aspect of this work. At the same time, future work should also consider the applicability and appropriateness of the diagnostic constructs used in these measures when applied in diverse populations. Lastly, in addition to ongoing work investigating country-level differences, further analysis and studies should also evaluate within-country differences to better explore these variations. We encourage more granular levels of data collection (e.g., differences in cases based on the urbanicity of their domicile), that might add nuance into these findings.
